# Prevalence and Clinical Parameters Associated With Chronic Total Occlusions in Patients With Chronic Coronary Syndromes: Insights From a Nationwide Registry

**DOI:** 10.1002/hsr2.70583

**Published:** 2025-03-18

**Authors:** Armin Attar, Mehrab Sayadi, Alireza Hosseinpour, Emmanouil S. Brilakis, Fereshte Mehdizade, Zahra Namvar, Alireza Khosravi, Maryam Boshtam, Feridoun Noohi, Ahmadreza Assareh, Toba Kazemi, Hossein Farshidi, Arsalan Khaledifar, Maryam Abbaszadeh, Nizal Sarrafzadegan

**Affiliations:** ^1^ Department of Cardiovascular Medicine Shiraz University of Medical Sciences Shiraz Iran; ^2^ Cardiovascular Research Center, Shiraz University of Medical Sciences Shiraz Iran; ^3^ School of Medicine, Shiraz University of Medical Sciences Shiraz Iran; ^4^ Minneapolis Heart Institute and Minneapolis Heart Institute Foundation Minneapolis Minnesota, USA; ^5^ Hypertension Research Center, Cardiovascular Research Institute, Isfahan University of Medical Sciences Isfahan Iran; ^6^ The Iranian Network of Cardiovascular Research (INCVR) Isfahan Iran; ^7^ Isfahan Cardiovascular Research Center, Cardiovascular Research Institute, Isfahan University of Medical Sciences Isfahan Iran; ^8^ Rajaie Cardiovascular Medical and Research Center, Iran University of Medical Sciences Tehran Iran; ^9^ Atherosclerosis Research Center, Ahvaz Jundishapur University of Medical Sciences Ahvaz Iran; ^10^ Cardiovascular Diseases Research Center, Birjand University of Medical Sciences Birjand Iran; ^11^ Department of Cardiology School of Medicine and Modeling in Health Research Center, Shahrekord University of Medical Sciences Shahrekord Iran; ^12^ Department of Clinical Sciences School of Medicine, Imam Hossein Hospital, Shahroud University of Medical Sciences Shahroud Semnan Iran

**Keywords:** chronic coronary syndrome, chronic total occlusion, coronary artery disease, prevalence

## Abstract

**Background and Aims:**

The prevalence of coronary chronic total occlusions (CTO) among patients with chronic coronary syndrome (CCS) and their associations with clinical factors have received limited study. We analyzed a national database registry to determine the prevalence, location, and parameters associated with coronary CTOs.

**Methods:**

We identified all CCS patients without prior coronary artery bypass graft surgery (CABG) who underwent coronary angiography in the Persian CardioVascular Disease Registry (PCVDR). We compared the baseline demographics and characteristics of patients with vs. without at least one CTO lesion. We used logistic regression analysis to identify parameters associated with coronary CTOs.

**Results:**

Among the 40,161 patients with CCS who underwent coronary angiography between March 2019 and December 2023, 6805 (17.86%) had at least one CTO. CTO patients were significantly older (64.43 ± 8.96 years vs. 62.64 ± 9.54 years, *p* < 0.001) and more likely to be men (75.3% vs. 54.4%, *p* < 0.001). The left anterior descending artery (70.4%) and right coronary artery (16.5%) were the most common CTO lesion locations. Older age (adjusted odds ratio [aOR] 95% confidence intervals [CI] 1.024 (1.021–1.028), male gender (aOR 2.865 (2.685–3.058), any smoking (aOR 1.256 (1.145–1.378), diabetes mellitus (aOR 1.372 (1.288–1.460), and dyslipidemia (aOR 1.166 (1.096–1.239) were independently associated with the presence of a CTO.

**Conclusion:**

Approximately 1 in 5 CCS patients without prior CABG undergoing coronary angiography in this national database registry had a CTO. Advanced age, male gender, history of smoking, diabetes mellitus, and dyslipidemia were associated with higher likelihood of coronary CTOs.

## 1 Introduction

Chronic Total Occlusion (CTO) represents a significant and complex aspect of coronary artery disease (CAD), especially among patients with chronic coronary syndrome (CCS). A CTO is defined as a complete blockage in a coronary artery with no antegrade blood flow through the lesion (thrombolysis in myocardial infarction (TIMI) grade 0 flow), lasting for at least 3 months, despite the presence of bridging collaterals that may supply the downstream vessel [1]. The incidence of CTO is estimated to be approximately 20% of patients with coronary artery disease in database registries [2, 3]. Severe calcifications, particularly in patients with chronic kidney disease or heightened cardiovascular risk profiles, have been shown to significantly increase procedural complexity and reduce success rates in CTO percutaneous coronary intervention (PCI), emphasizing the importance of tailored interventional strategies in this subset of patients [4]. Understanding the incidence and the associated clinical parameters is essential due to its implications for patient management, clinical outcomes, and healthcare resource distribution.

The high prevalence of CTOs underscores the need for effective diagnostic and therapeutic strategies. Advances in imaging modalities, including intravascular ultrasound (IVUS) and optical coherence tomography (OCT), have significantly improved procedural planning and outcomes in CTO PCI [5]. Additionally, the integration of novel approaches and emerging technologies, such as hybrid imaging and artificial intelligence, offers new possibilities for personalized treatment of CTOs [6].

We examined the prevalence and location of CTOs among CCS patients undergoing coronary angiography in a national database registry and identified clinical parameters associated with the presence of a CTO.

## 2 Methods

### 2.1 Study Design and Population

We examined the Persian CardioVascular Disease Registry (PCVDR), a prospective national database implemented in 2017. This registry collects data on coronary angiography and PCI from 19 experienced centers in Iran, gathering detailed information on baseline demographics, characteristics of angiography and PCI, and follow‐up outcomes for patients undergoing non‐emergent coronary interventions [7]. For this study, all patients with CCS without prior coronary artery bypass graft surgery (CABG) and without previous history of acute coronary syndrome (ACS) undergoing coronary angiography for the first time were considered eligible. As the main aim of the study was to evaluate the incidence of CTO lesions in patients with newly de novo diagnosed CCS, all patients with a prior diagnosis of CAD, including those who had undergone CABG, were excluded from this study. The participants included were stratified based on the presence of a CTO. Patients who had not undergone coronary angiography, missing angiography details, and any other condition except CCS (e.g., ACS) as their primary diagnosis were excluded from the analysis. The protocol for this study was approved by the institutional review board of the Shiraz University of Medical Sciences (IR.SUMS.MED.REC.1402.402). The database of the present study was anonymized. The patients agreed on the possible use of their anonymized data for research purposes. The present study was conducted in accordance to the principles of the Declaration of Helsinki.

### 2.2 Definitions and Endpoints

Coronary CTO was defined as complete occlusion of the coronary artery with no forward blood flow (TIMI grade 0 flow) through the lesion with a presumed or documented duration of at least 3 months [1]. CCS encompasses various clinical syndromes that result from structural or functional changes associated with chronic coronary artery and/or microcirculatory disease. These changes can cause an imbalance between myocardial oxygen demand and blood supply, leading to transient and reversible ischemia, often triggered by physical exertion, emotional stress, or other stimuli. This ischemia may present as angina, chest discomfort, dyspnea, or remain asymptomatic. While these conditions can remain stable for extended periods, they are often progressive and have the potential to destabilize suddenly, leading to the onset of ACS [8]. Furthermore, the definition of CCS falls within several clinical scenarios: (i) suspected CAD with stable angina or dyspnea; (ii) new heart failure or left ventricular dysfunction with suspected CAD; (iii) symptomatic or asymptomatic patients within a year of an ACS or recent revascularization; (iv) symptomatic or asymptomatic patients over a year after diagnosis or revascularization; (v) angina with suspected vasospastic or microvascular disease; and (vi) asymptomatic individuals where CAD is detected during screening [9]. A successful PCI for CTO was defined as post‐PCI TIMI grade 3 flow and < 50% residual stenosis in the vessel [10].

Among the eligible CCS patients with available angiography data, patients were divided into two groups with and without CTO. The coronary arteries with a lesion (≥ 50% stenosis in the artery's lumen) were compared between groups based on the number of vessels involved (single [SVD), two [2VD], and three‐vessel disease [3VD]) and the anatomical distribution of the stenoses. The decision to perform revascularization using PCI or CABG was mainly based on the most recent European Society of Cardiology (ESC) Guidelines [8, 9] and also the patients' preference whether to accept the CABG procedure or not.

### 2.3 Statistical Analysis

Continuous variables were presented as mean ± standard deviation using independent samples *t*‐test or Mann–Whitney *U* test, as appropriate. Categorical variables were presented as frequencies and percentages and compared using the *χ* ^2^ test. We performed a multivariable logistic regression analysis to identify parameters associated with the presence of a CTO. The backward stepwise method was used by entering all the candidate variables into the model and iteratively removing the least significant ones. This process continued until all the remaining variables were statistically significant. A *p‐*value of 0.05 was also set as the threshold of significance. The analyses were performed using SPSS statistical software version 26.0 or R Software version 4.3.2.

## 3 Results

**Figure 1:**
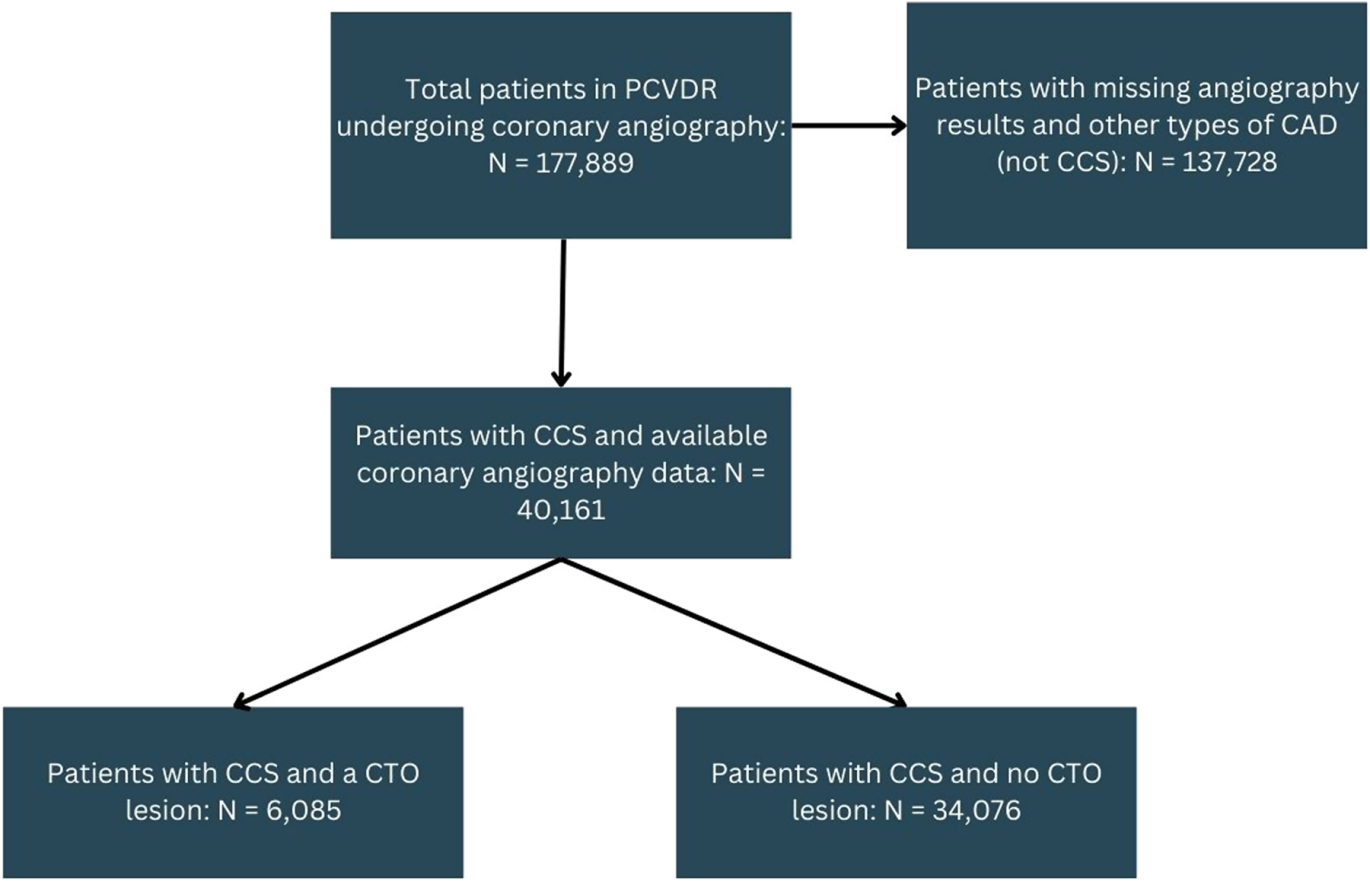
Flowchart of the patient selection. CAD, coronary artery disease; CCS, chronic coronary syndrome; CTO, chronic total occlusion; PCVDR, Persian CardioVascular Disease Registry.

A coronary CTO was present in 6085 of 40,161 CCS patients without prior CABG who underwent coronary angiography during the study period (17.86%, 95% confidence intervals [CI] 17.45–18.27) (Figure 1). CTO patients were significantly older (64.43 ± 8.96 vs. 62.64 ± 9.54 years, *p* < 0.001) and more likely to be men (75.3% vs. 54.4%, *p* < 0.001), and to have a smoking history (11.4% vs. 7.4%, *p* < 0.001), diabetes mellitus (33.0% vs. 27.7%, *p* < 0.001), dyslipidemia (34.7% vs. 30.5%, *p* < 0.001), end‐stage renal disease (ESRD) (0.4% vs. 0.2%, *p* = 0.003), and a history of cardiac arrest (0.03% vs. 0.00%, *p* = 0.02) compared with patients without a CTO lesion. CTO patients were less likely to have chronic kidney disease (CKD) (0.1% vs. 0.6%, *p* = 0.002), atrial fibrillation (AF) (0.2% vs. 0.5%, *p* = 0.009), and arrhythmia (0.2% vs. 0.4%, *p* = 0.01) compared with no CTO group (Table 1).

**Table 1:** General characteristics and previous cardiovascular history.

Characteristics	Patients with a CTO lesion (*n* = 6085)	Patients without a CTO lesion (*n* = 34,076)	***p*‐value**
Age	64.43 ± 8.96	62.64 ± 9.54	< 0.001
Gender, male	4962 (75.3)	19,569 (54.4)	< 0.001
Smoking (tobacco)	696 (11.4)	2527 (7.4)	< 0.001
Alcohol consumption	24 (0.4)	84 (0.2)	0.05
Diabetes mellitus	2006 (33.0)	9452 (27.7)	< 0.001
Family history of cardiovascular diseases	1113 (18.3)	6035 (17.7)	0.30
Cerebrovascular disease	31 (0.5)	145 (0.4)	0.40
Hypertension	3086 (50.7)	16,850 (49.5)	0.07
Heart failure	19 (0.3)	71 (0.2)	0.10
Dyslipidemia	2109 (34.7)	10,401 (30.5)	< 0.001
Peripheral arterial disease	6 (0.1)	13 (0.03)	0.06
Non‐coronary heart surgery	7 (0.1)	17 (0.04)	0.08
CKD	61 (0.1)	220 (0.6)	0.002
ESRD	27 (0.4)	79 (0.2)	0.003
Atrial fibrillation	14 (0.2)	160 (0.5)	0.009
Arrhythmia	11 (0.2)	129 (0.4)	0.02
VHD	27 (0.4)	193 (0.6)	0.20
History of cardiac arrest	2 (0.03)	0 (0.00)	0.02
Asymptomatic	249 (57.2)	1512 (56.2)	0.70
History of performed noninvasive imaging studies	6017 (98.9)	33,873 (99.4)	< 0.001

*Note:* Data are presented as either mean ± standard deviation or frequency (percentage).

Abbreviations: CKD, chronic kidney disease; CTO, chronic total occlusion; ESRD, end‐stage renal disease; VHD, valvular heart disease.

**Figure 2:**
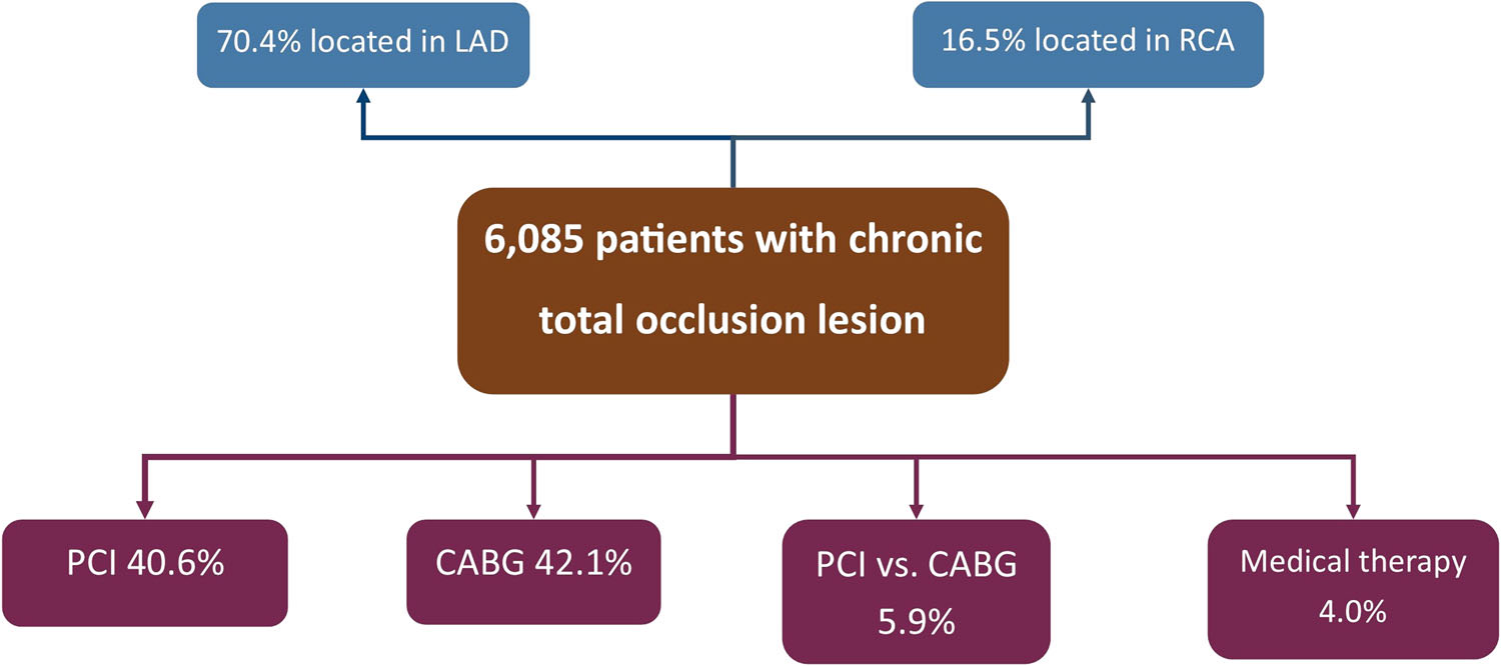
Flowchart showing the anatomical distribution of the CTO lesions and the recommended therapeutic strategy of the registry patients. CABG, coronary artery bypass grafting; CTO, chronic total occlusion; LAD, left anterior descending; PCI, percutaneous coronary intervention; RCA, right coronary artery.

Compared with the no CTO group, CTO patients were more likely to have two‐vessel (28.5% vs. 15.3%, *p* < 0.001), or three‐vessel (51.6% vs. 10.0%, *p* < 0.001) coronary artery disease, or a left main lesion (7.2% vs. 2.2%, *p* < 0.001) and less likely to have SVD (19.0% vs. 23.2%, *p* < 0.001) or minimal CAD (0.0% vs. 21.4%, *p* < 0.001). The location of CTO lesions and the recommended therapeutic strategy are shown in Table 2 and Figure 2.

**Table 2:** The location of the CTO lesion stratified by the artery and branch site.

Vessel name	*N* (%)	Branch name	*N* (%)
LAD	4287 (70.4%)	Proximal LAD	2063 (33.9)
		Midpart LAD	2174 (35.7)
		Distal LAD	46 (0.8)
LCX	433 (5.5%)	Proximal LCX	183 (3.0)
		Distal LCX	151 (2.5)
RCA	1013 (16.5%)	Proximal RCA	209 (3.4)
		Distal RCA	794 (13.1)
Diagonal	141 (2.3%)	Diagonal 1	132 (2.2)
		Diagonal 2	9 (0.1)
OM	163 (2.7%)	OM 1	129 (2.1)
		OM 2	28 (0.5)
		OM 3	5 (0.1)
PDA	3 (0.03)		
PLV	1 (0.01)		
Ramus	101 (1.7)		

Abbreviations: LAD, left anterior descending; LCX, left circumflex; OM, obtuse marginal; PDA, posterior descending artery; PLV, posterior left ventricular; RCA, right coronary artery.

On multivariable analysis older age (aOR [95% CI] 1.024 [1.021–1.028]), male gender (aOR [95% CI] 2.865 [2.685–3.058]), any smoking (aOR [95% CI] 1.256 (1.145 –1.378)), diabetes mellitus (aOR [95% CI] 1.372 [1.288–1.460]) and dyslipidemia (aOR [95% CI] 1.166 [1.096–1.239]) were independently associated with the presence of a CTO (Table 3).

**Table 3:** Multivariable logistic regression analysis of parameters associated with presence of a coronary CTO.

Variable	aOR	95% CI	*p*‐value
Age	1.024	1.021–1.028	< 0.001
Gender, male	2.865	2.685–3.058	< 0.001
Smoking	1.256	1.145–1.378	< 0.001
Diabetes mellitus	1.372	1.288–1.460	< 0.001
Dyslipidemia	1.166	1.096–1.239	< 0.001

Abbreviations: aOR, adjusted odds ratio; CI; confidence interval.

CTO patients were more likely to undergo PCI (40.6% vs. 28.9%, *p* < 0.001), CABG (42.1% vs. 6.9%, *p* < 0.001), or CABG vs. multivessel PCI (based on the patient's preference) (5.9% vs. 1.1%, *p* < 0.001), whereas and less likely to undergo medical treatment without revascularization (4.0% vs. 5.5%, *p* < 0.001; Table 4).

**Table 4:** The angiographic findings and the recommended therapeutic strategy among the groups.

		Patients with a CTO lesion	Patients without a CTO lesion	*p*‐value
Angiographic finding	Minimal CAD	0 (0.0)	7282 (21.4)	< 0.001
	Indeterminate	0 (0.0)	671 (2.0)	< 0.001
	SVD	1150 [11]	7912(23.2)	< 0.001
	2VD	1737 (28.5)	5230 (15.3)	< 0.001
	3VD	3142 (51.6)	3400 (10.0)	< 0.001
	LM lesion	440 (7.2)	739 (2.2)	< 0.001
Recommended therapeutic strategy	PCI	2470 (40.6)	9846 (28.9)	< 0.001
	CABG	2560 (42.1)	2340 (6.9)	< 0.001
	CABG vs. multivessel PCI	356 (5.9)	364 (1.1)	< 0.001
	Medical treatment without prompt revascularization	244 (4.0)	1884 (5.5)	< 0.001

*Note:* Data are presented as frequency (percentage).

Abbreviations: CABG, coronary artery bypass grafting; CAD, coronary artery disease; CTO, chronic total occlusion; LM lesion, left main lesion; PCI, percutaneous coronary intervention; SVD, single vessel disease; 2VD, two‐vessel disease; 3VD, three‐vessel disease.

Data on subsequent PCI after the recommended strategy were limited, with information available for only 434 patients with CTO lesions. The mean contrast volume and radiation dose were 227.22 ± 158.26 cc and 1294.78 ± 1337.19 mGy, respectively. Most patients were accessed via the femoral artery (82.5%), followed by the right radial artery (12.4%) and the left radial artery (1.6%). Complications were rare, with significant dissections occurring in 2.7% of cases and perforations in 0.9%. After PCI, 89.8% of patients achieved a TIMI grade 3 flow. The rates for TIMI flow grades 0, 1, and 2 were 8.7%, 1.0%, and 0.5%, respectively, following revascularization.

## 4 Discussion

The main findings of our study are: (a) CTOs were present in 17.86% of CCS patients undergoing coronary angiography without prior history of CABG; (b) most CTOs were located in the LAD; (c) and older age, male gender, smoking, diabetes mellitus, and dyslipidemia were associated with higher likelihood of having a CTO.

The prevalence of CTO reported in the literature ranges from approximately 6% among all the PCI procedures up to 52% in the whole population of CAD patients with available angiography results (Table 5) [3, 12, 13, 14, 15, 16, 17]. The prevalence of CTO in our study was 17.86%. Previous studies on CTO prevalence included all patients undergoing PCI, with many having a history of CABG or PCI [3, 12]. However, there is limited research on CTO prevalence among patients with CCS who have no history of acute coronary events. Our study, which comprised over 40,000 patients with CCS, provides valuable insight into the prevalence of CTO in this specific population. In prior studies the most common CTO location is in the RCA followed by the LAD [12, 18]. In contrast most CTOs in our study were located in the LAD (70.4%), while only 16.5% located in the RCA. The discrepancy between the previous research and our study may stem from different factors including population differences, genetic and ethnic factors, and temporal changes. Also, a significant proportion of patients with CTO did not undergo revascularization and were treated with medical therapy [12, 18], whereas our results demonstrated that about 85% of our patients identified with a CTO lesion were advised to undergo revascularization (PCI or CABG). The higher prevalence of CTOs in the LAD artery and the fact that LAD subtends large areas of myocardial territory may underpin the higher rate of revascularization in our study compared with other studies. Regarding the relationship between the extent of CAD and LVEF, it has been previously demonstrated that the extent of coronary atherosclerosis is linked to subclinical reductions in LVEF, even in patients with preserved ejection fraction [19]. Additionally, the potential benefits of CTO revascularization have been well‐documented, reporting significant improvements in LVEF and clinical outcomes following successful revascularization [11]. The potential underlying mechanisms resulting in left ventricular function recovery following CTO revascularization include enhanced myocardial perfusion, reduction of ischemic burden, and improvement in collateral circulation [20].

**Table 5:** Prevalence of CTO and characteristics of the studied populations in previous studies.

Study	The studied population	Prevalence of CTO
van Veelen et al. (the Netherland Heart Registration) [3]	Among all the performed PCI procedures	6.3%
Fefer et al. (the Canadian Multicenter Chronic Total Occlusions Registry) [12]	Among all the patients undergoing coronary angiography	18.2%
Christofferson et al. [13]	Based on the available diagnostic coronary angiography data from patients with suspected or known CAD at a single‐center hospital	52%
Jeroudi et al. [14]	Based on the diagnostic coronary angiography data from patients with known CAD at a single‐center hospital	46%
Tsai et al. (the VA CART Program) [15]	All the patients with obstructive CAD found during coronary angiography	26.4%
Tomasello et al. (the Italian Registry of Chronic Total Occlusions) [16]	Patients undergoing coronary angiography with no previous CABG	13.3%

Abbreviations: CABG, coronary artery bypass grafting; CAD, coronary artery disease; CTO, chronic total occlusion; PCI, percutaneous coronary intervention; VA CART; US Department of Veterans Affairs Clinical Assessment Reporting and Track.

The results from the multivariable logistic regression analysis identified several clinical parameters independently associated with CTO lesions among patients with chronic coronary syndromes. Age, gender, smoking, diabetes mellitus, and dyslipidemia were all significantly associated with the presence of CTO lesions. Among the recognized predictors, gender demonstrated a moderate association and the rest of the predictors had weak association. Specifically, each year increase in age was associated with a 2.4% increase in the odds of having a CTO lesion. Men were 2.87 times more likely to have a CTO lesion compared with women. Smoking increased the odds of CTO by 25.6%, while diabetes mellitus increased the odds by 37.2%. Dyslipidemia was also a significant parameter, with a 16.6% increase in the odds of CTO. Although the risk factors for coronary artery disease are well‐established and have been extensively studied, the current research is less focused on clinical characteristics associated with the presence of CTO and the potential risk factors of CTO lesions among patients with CCS are less understood. Our findings align with those reported in other studies on predictors of CAD. Age, male gender, smoking, diabetes mellitus, and dyslipidemia are among the conventional and well‐established risk factors for CAD. Previous research has shown that traditional risk factors like smoking, diabetes, and dyslipidemia significantly contribute to the risk of coronary heart disease [21].

Our study has limitations. The retrospective design of this investigation may introduce confounding and bias and cannot establish causality. Hence, future randomized trials and propensity‐matched studies are warranted to assess the long‐term outcomes and compare different treatment strategies. The advised therapeutic strategy was missing for some patients especially the group without CTO and this may have had an impact on the results. The clinical outcomes including major adverse cardiovascular events and mortality data were not available for this study, hence comparison between the groups and time‐to‐event analysis were not applicable. Data on subsequent revascularization methods were incomplete, and the available information may be subject to potential bias. Although data on obesity was unavailable, a correlation between dyslipidemia and obesity can be inferred, as patients with obesity exhibit dyslipidemia at rates three times higher than the general population [22]. Consequently, a potential link between obesity and CTO may exist, though further studies are required to confirm this. This explanation was added to the limitations section. The clinical variables associated with CTO were identified in the subset of patients with CCS and no previous history of acute coronary events. The results may not be generalized to all the patients with CAD and previous history of acute myocardial infarction.

In conclusion, the prevalence of patients with at least one CTO lesion was estimated to be 17.86% in the population of patients with CCS. The primary location of CTO lesions was in LAD artery followed by RCA. The majority of patients with CTO (88.6%) underwent coronary revascularization (PCI or CABG) and the procedural success was estimated to be 89.8% based on the available data. Advanced age, male sex, smoking, diabetes mellitus, and dyslipidemia were associated with the presence of CTO in patients with CCS.

## Author Contributions

**Armin Attar:** conceptualization, methodology, data curation, investigation, writing – review and editing, writing – original draft, project administration, resources, supervision. **Mehrab Sayadi:** formal analysis, data curation, software. **Alireza Hosseinpour:** conceptualization, writing – original draft, writing – review and editing, visualization, software. **Emmanouil S. Brilakis:** writing – review and editing. **Fereshte Mehdizade:** writing – original draft. **Zahra Namvar:** writing – original draft. **Alireza Khosravi:** writing – original draft, methodology, conceptualization. **Maryam Boshtam:** conceptualization, writing – original draft, methodology. **Feridoun Noohi:** conceptualization, writing – original draft, methodology. **Ahmadreza Assareh:** conceptualization, writing – original draft, methodology. **Toba Kazemi:** conceptualization, writing – original draft, methodology. **Hossein Farshidi:** conceptualization, writing – original draft, methodology. **Arsalan Khaledifar:** conceptualization, writing – original draft, methodology. **Maryam Abbaszadeh:** conceptualization, writing – original draft, methodology. **Nizal Sarrafzadegan:** conceptualization, writing – original draft, methodology, supervision, validation, resources, project administration.

## Conflicts of Interest

Dr. Brilakis discloses the following relationships: consulting/speaker honoraria from Abbott Vascular, American Heart Association (associate editor: Circulation), Biotronik, Boston Scientific, Cardiovascular Innovations Foundation (Board of Directors), CSI, Elsevier, GE Healthcare, IMDS, Medtronic, and Teleflex; research support: Boston Scientific, GE Healthcare; owner, Hippocrates LLC; shareholder: MHI Ventures, Cleerly Health, Stallion Medical. The other authors declare no conflicts of interest.

## Transparency Statement

The lead author Alireza Hosseinpour affirms that this manuscript is an honest, accurate, and transparent account of the study being reported; that no important aspects of the study have been omitted; and that any discrepancies from the study as planned (and, if relevant, registered) have been explained.

## Data Availability

The data that support the findings of this study are available from the corresponding author upon reasonable request.
